# Annotations

**Published:** 1897-08-28

**Authors:** 


### Annotations.

### In Praise of Vestries.

It is a pity and an injustice that the word vestryman should have come to signify in the popular mind the incarnation of narrow-minded pig-headedness. The vestry is a narrow area, and it is not the vestryman's business, *quâ* vestryman, to look outside its borders. It is his duty to take only parochial views, and doubtless there may be occasions when these lead him, in the interests of his own little bourg, to distrust the actions of his neighbours. But the very smallness of the area a vestry controls has an advantage which the advocates of centralized administration forget. Social experiments of every kind can more easily be made on a small scale than a large, and comparison of results can more reasonably be made between neighbouring districts than between cities far apart. The advantages gained by the subdivision of London into vestries can be suggested by one instance. It is usual to sprinkle the streets in summer, not with water alone but with disinfectants. If two neighbouring districts try, say, the one carbolic acid, the other Condy's fluid for a season, it is easy for the two vestries to compare notes as to results. "Our death-rate was unaltered";—"Our death-rate decreased by so much." When such facts as these are brought forward it is easy to decide which disinfectant is the more efficacious, and the conclusion is more reasonable than if the experiments were tried all over London in successive years, when climatic conditions are very different; or if, simultaneously, London and Manchester made the experiment, with differences of soil, climate, density of population, and other things to be considered in judging the result. The vestry as a compact entity, knowing its own ground as no larger body can know its wider area, can test an idea with a completeness almost impossible in a larger sphere. We of this age tend to generalise too much, especially in our social experiments, and it is well to remember, to the honour of little things, that the little states of Greece, trying various forms of government on their little scale, have been through subsequent ages the reference-book on government of the world.

### Who Are the Dirtiest Men?

"I suppose your inmates are pretty dirty," remarked a Hospital representative to the superintendent of a lodging-house, looking at the dockers who lounged around, smoking and playing draughts or dominoes. "These aren't the dirtiest kind," was the reply. "Their clothes are dirty, and perhaps their hands ingrained through rough work; but they are nothing to the broken-down gentleman, or man who sets up for being a gentleman, for personal filthiness. I know those gentry—the kind with a collar, a necktie perhaps, and the coat tightly buttoned across. No waistcoat, you may be sure, and probably no shirt either. They have large airs, these gentry! 'I want your best bed!' as if they were coming into a first-class hotel! At first they aren't so straight as that; they have excuses—explanations that deceive nobody—for their coming to a common lodging-house at all. They have missed the last train to their suburban home, and 'just out of curiosity, you know,' have come here because they would like 'to know how these places are managed.' Or they explain that they are on the staff of some journal, and are merely investigating the character of lodging-houses on its behalf." Here The Hospital man blushed, but recovered leisurely on reflecting that a *bonâ fide* journalist, bent on *bonâ fide* personal investigation, does not reveal his status or mission to the manager. "And I know all the time," concluded the manager with some scorn, "that they haven't a copper in their pockets. Drink is written all over them. They're hopeless creatures, who can't do a day's work, and live simply by begging of one kind or another. As they come down in the world they lose heart more and more, and at last they haven't even as much self-respect as serves to make them want to be clean. They've the chance here—a bath and hot and cold water at their command, and nothing to pay for it; but they don't want to be clean, and it is their beds that want the most careful airing." The pity of it! thought the listener, that those who once stood comparatively high should sink thus to be lower than the lowest. But is it not merely a sign of that weakness of nature, that lax hold of what was good and pure in every relation of life, in every department of work, which has brought them where they are now? "Cleanliness is next to godliness," says the proverb, and it is an integral part of self-respect.

### Young Criminals at Home and Abroad.

The Hon. Robert White, while imprisoned in Holloway Gaol in consequence of his share in the Jameson Raid, was grieved at the treatment accorded to young prisoners. It seemed that even while they were unconvicted they were subjected to much the same treatment as older and proved criminals; shut up in cells in solitude and allowed out only for a brief space of formal exercise daily. On regaining his freedom Mr. White took occasion to inquire how children accused of crime are treated in Paris. He finds that every consideration is shown for their possible innocence, and that they are carefully guarded from contact with criminals of maturer years. The first inquiries take place, not before an open Court, but in presence only of a *juge d'instruction*, his clerk, and one or two policemen, and the inquiry is carried on in a kindly and familiar tone which elicits truth without frightening the accused. Boy prisoners are kept in a prison by themselves; girls, in a wing entirely apart from their seniors, in the women's prison. Up to the age of twelve they occupy cells with doors open, that the terror which solitude causes in young children may not afflict them, though a warder patrols the corridor to prevent communication. Even when they are of an age which makes the authorities consider it advisable to lock them in, the cells are cheerful and airy, and besides receiving visits and instruction from a schoolmaster, they are given work to do of a simple yet interesting kind. It certainly seems as if, in the case of children not yet convicted of crime, it is cruel to treat them as if they were proved guilty, as Mr. White seems to think is the case; and education and the interest of learning some kind of work will do much to counteract the evil effects of solitude. But perhaps these effects are less serious than Mr. White imagines. He admits that the sobbing he heard at night in the cells at Holloway was often a ruse on the part of "artful dodgers." The children who get into gaol are largely recruited from the criminal class itself, and while one would gladly show any kindness that would induce these children to adopt an honourable life, we are not prepared to say that the present system directly tends to make the innocent take to crime.

